# Vascular Waveform Analysis of Flap-Feeding Vessels Using Color Doppler Ultrasonography

**DOI:** 10.1155/2014/249670

**Published:** 2014-04-07

**Authors:** Akihiro Ogino, Kiyoshi Onishi

**Affiliations:** Department of Plastic and Reconstructive Surgery, 6-11-1, Omori-nishi, Ota-ku, Tokyo 143-8541, Japan

## Abstract

We performed vascular waveform analysis of flap-feeding vessels using color Doppler ultrasonography and evaluated the blood flow in the flaps prior to surgery. Vascular waveform analysis was performed in 19 patients. The analyzed parameters included the vascular diameter, flow volume, flow velocity, resistance index, pulsatility index, and acceleration time. The arterial waveform was classified into 5 types based on the partially modified blood flow waveform classification reported by Hirai et al.; in particular, D-1a, D-1b, and D-2 were considered as normal waveforms. They were 4 patients which observed abnormal vascular waveform among 19 patients (D-4 : 1, D-3 : 1, and Poor detect : 2). The case which presented D-4 waveform changed the surgical procedure, and a favorable outcome was achieved. Muscle flap of the case which presented D-3 waveform was partially necrosed. The case which detected blood flow poorly was judged to be the vascular obstruction of the internal thoracic artery. In the evaluation of blood flow in flaps using color Doppler ultrasonography, determination of not only basic blood flow information, such as the vascular distribution and diameter and flow velocity, but also the flow volume, vascular resistance, and arterial waveform is essential to elucidate the hemodynamics of the flap.

## 1. Introduction

For the evaluation of blood flow in flap-feeding vessels in the field of plastic and reconstructive surgery, various methods are used, such as angiography, MRA, and MDCT, and stable results are obtained; but these methods can be confirmed the presence and distribution of a flap-feeding vessels, and the details, such as the flow velocity and flow volume and vascular resistance, cannot be evaluated. Since color Doppler ultrasonography can be visualized noninvasively and easily, relative low cost of the microblood vessels has recently been used to identify flap-feeding vessels, particularly perforating arteries, and its usefulness has been reported [1–3]. We performed vascular waveform analysis of flap-feeding vessels using color Doppler ultrasonography and evaluated the blood flow in the flaps prior to surgery. In the present study, we introduce an overview of the tests and report on its usefulness.

## 2. Subjects and Methods 

Vascular waveform analysis was performed in 19 patients who underwent reconstructive surgery with pedicle flap (9 patients males and 10 patients females; age was 33∼79; mean age was 59 years). Primal disease was 7 patients of mediastinitis, 7 patients of malignant tumor, 2 patients of esophagus skin fistula, 1 patient of scar contracture after burn, 1 patient of abdominal wall incisional herniatis, and 1 patient of vagina defect. Transplanted flap was 8 patients of latissimus dorsi musculocutaneous flap, 6 patients of rectus abdominis musculocutaneous flap, 3 patients of pectoralis major musculocutaneous flap, and 2 patients of tensor fascia lata musculocutaneous flap (Table 1).

No underlying severe disease, such as diabetes mellitus, atherosclerosis obliterans, or chronic renal disease, was observed in any patient.

The XARIO SSA-660A-770A (Toshiba) as an ultrasonic diagnosis system and a high frequency linear surface probe (7.5–10 MHz) was used.

Concerning items of vascular waveform analysis, the vascular diameter, flow volume (F-V), maximum flow velocity (*V*_max⁡_), minimum flow velocity (*V*_min⁡_), mean flow velocity (*V*_mean_), and resistance index (RI), pulsatility index (PI), and acceleration time (AT: time reaching a peak during systole) based on arterial waveforms were measured. F-V was calculated by multiplying the time integral value of *V*_mean_ by the cross-sectional area of the vessel assumed to be a circle. RI was calculated as (*V*_max⁡_ − *V*_min⁡_)/*V*_max⁡_ and PI as (*V*_max⁡_ − *V*_*min*_)/*V*_mean_. The vascular waveform was measured at a site of the flap-feeding vessels after branching within 3 cm. The incidence angle of ultrasound during vascular waveform analysis was ≤60° in principle.

Blood flow evaluation of flap feeding vessels was performed referring to the standard value of superior epigastric artery, deep inferior epigastric artery, subscapular artery, and thoracodorsal artery which we set up before [4] (Table 2).

**Figure 1:**
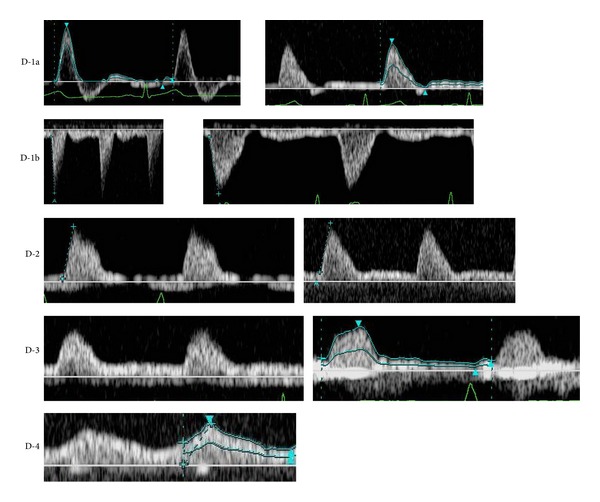
Novel classification of the vascular waveform of flap-feeding vessels in Doppler ultrasonography. D-1a type: normal waveforms in which systolic crests rise steeply, followed by reflux components; D-1b type: normal waveforms in which systolic crests rise steeply but reflux components are lost, and a notch is noted between systolic and diastolic waves; D-2 type: peaks are formed, but the width of systolic crests is wider than normal, reflux components are lost, and no notch is present between systolic and diastolic waves; D-3 type: systolic crests are moderate and form no peak; and D-4 type: moderate continuous waveforms.

Arterial waveforms were classified into the following 5 types using a partially modified blood flow waveform classification reported by Hirai et al. [5] (Figure 1).

D-1a, D-1b, and D-2 waveforms were regarded as normal; D-3 and D-4 were regarded as abnormal.

Moreover, venous blood flow was confirmed by visualize the running of the vessel and venous waveform.

This study was approved by the Ethics Committee of Toho University Omori Medical Center (number: 24026) and performed after obtaining comprehension from the patients with sufficient informed consent.

## 3. Results 

**Figure 2:**
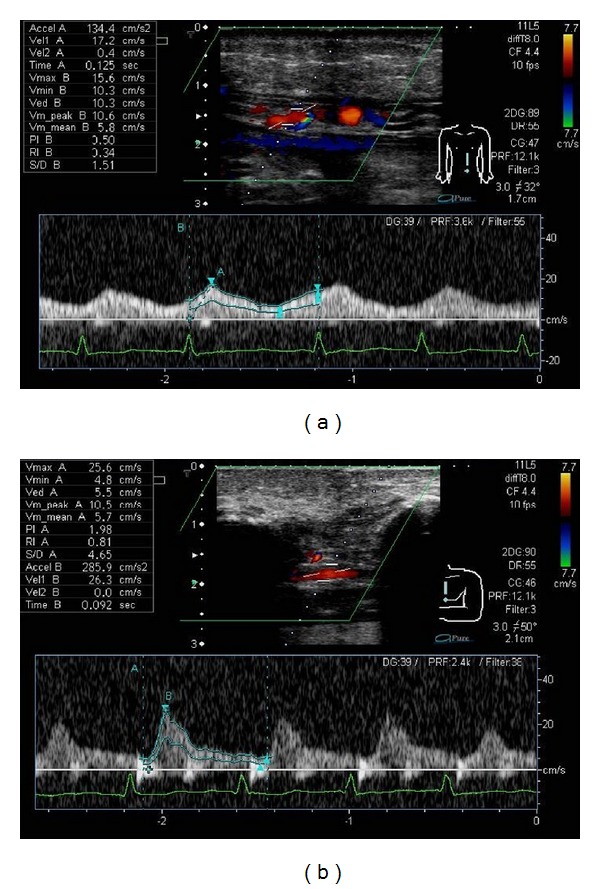
(a) Patient 1. Waveform of the superior epigastric artery (Type D-4). The *V*_max⁡_ was 15.6 cm/sec, the lower limit of the standard value, and RI and PI were 0.34 and 0.50, respectively, apparently lower than the standard values. (b) Patient 1. Waveform of the internal thoracic artery (Type D-2). The waveform was a normal waveform.

**Figure 3:**
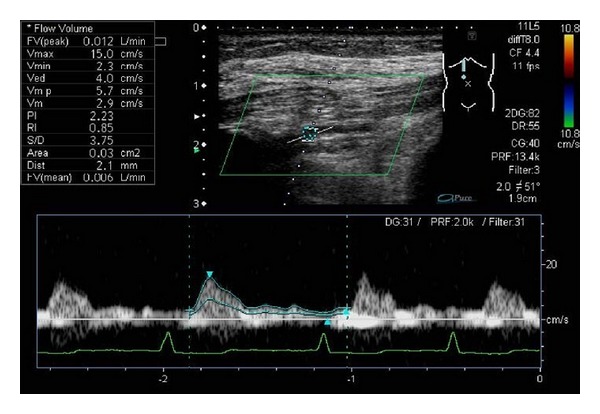
Patient 10. Waveform of the superior epigastric artery (Type D-3). The rectus abdominis musculocutaneous flap was partially necrosed.

It was 4 patients which observed abnormal vascular waveform among 19 patients which performed vascular waveform analysis of flap-feeding vessels using color Doppler ultrasonography prior to surgery (D-4: 1 patient, D-3: 1 patient, and Poor detect: 2 patients). An abnormal D-4 waveform was noted in a case in which superior epigastric arterial waveform analysis was performed before surgery for sternal osteomyelitis (Patient 1). The *V*_max⁡_ was 15.6 cm/sec, being slower than the standard; RI was 0.34, and PI was 0.50, being lower than the standard. Since the waveform of internal thoracic artery was a normal waveform, a part of internal thoracic artery was damaged on the occasion of sternum debridement, and a possibility of angiostenosis was suspected (Figure 2). Therefore the surgical procedure was changed to one using latissimus dorsi musculocutaneous flap, and a favorable outcome was achieved. The case which presented D-3 waveform planned reconstruction by rectus abdominis muscle flap to mediastinitis and vascular waveform analysis of the superior epigastric artery was performed. However, the *V*_max⁡_ was 15.0 cm/sec, being lower than the standard, and the muscle flap was partially necrosed (Figure 3). The case which detected blood flow poorly planned reconstruction by rectus abdominis musculocutaneous flap to mediastinitis and vascular waveform analysis of the superior epigastric artery was performed. However blood flow detection of the internal thoracic artery to the superior epigastric artery was poor. The blood vessel which flows into rectus abdominis muscle was confirmed in the direction of the outside; we performed blood flow evaluation by vascular waveform analysis. Although the vessel diameter was slightly as thin as about 1 mm, the vascular waveform was normal as D-2 waveform. Therefore we judged that the flap transplantation was possible and performed reconstruction using rectus abdominis musculocutaneous flap which was planned. The flap was partially necrosed after the operation. It was guessed to be because the tension of the flap distal part was strong in order to cover the defect of the chest upper part. Patient 17 was judged to be the vascular obstruction of the internal thoracic artery and was changed into reconstruction using omentum and pectoralis major muscle flap. Other 15 patients were valued with sufficient blood flow volume and flow velocity, and the vascular waveform was also normal waveform and the flap completely survived (Table 3).

Pedicled rectus abdominis musculocutaneous flaps supplied by the superior epigastric artery and vein were transplanted in 4 patients. The waveform type was D-1b in 1 patient and D-2 in 2 patients and D-3 in 1 patient. One of 2 flaps prepared with D-2 waveform vessels was completely survived, but the other flap was partially necrosed. One flap prepared with D-3 waveform vessels was partially necrosed.

Pedicled rectus abdominis musculocutaneous flaps supplied by the deep inferior epigastric artery and vein were transplanted in 2 patients. The waveform type was D-1b in 1 patient and D-2 in 1 patient, and all flaps were completely survived.

Pedicled latissimus dorsi musculocutaneous flaps supplied by the thoracodorsal artery and vein were transplanted in 8 patients. The waveform type was D-1a in 3 and D-1b in 3 and D-2 in 2 patients, and all flaps were completely survived.

Pedicled pectralis major musculocutaneous flaps supplied by the thoracoacrominal artery and vein were transplanted in 3 patients. The waveform type was D-1b in 3 patients, and all flaps were completely survived.

Pedicled tensor fascia lata musculocutaneous flaps supplied by the lateral circumflex femoral artery and vein were transplanted in 2 patients. The waveform type was D-1b in 1 and D-2 in 1 patient, and all flaps were completely survived (Table 4).

All surgeries were performed by the same operator.

## 4. Discussion 

To plan reconstruction with a vascularized flap, it is necessary to investigate the hemodynamics of feeding vessels before surgery in consideration of individual differences, vascular mutation, angiostenosis, and vascular obstruction. For the evaluation of blood flow in flap-feeding vessels in the field of plastic and reconstructive surgery, various methods are used, such as Doppler probe, angiography, MRA, and MDCT [6, 7].

Doppler probe has many advantages, such as simple operation, low cost, and short time of inspection. On the other hand, since the depth of the detected blood vessel cannot be judged, there is a risk of detecting a blood vessel deeper or shallower than the target blood vessel by mistake [6]. Although angiography is suitable for blood-flow evaluation of medium sized artery, it has problems, such as high invasion, radioactive exposure, and a high cost. Moreover, the special technique of image processing method is needed for imaging a thin artery like perforating vessels. Since MRA and MDCT have high contrast resolution, not only blood vessels and bones can be visualized, but also muscles and the surrounding soft tissue. Moreover, a perforating vessel less than 1 mm [7] also can be visualized. Thus, although highly precise image is obtained by low invasion, condition setting is necessary in order to enable the imaging of perforating vessels, such as to squeeze the particular vessel and to reduce the slice thickness of the image. In addition, the available facilities are limited because equipment is expensive.

On the other hand, color Doppler ultrasonography can be visualized noninvasively and easily, relative low cost the micro blood vessels compared to these tests, it has recently been used to identify flap-feeding vessels, particularly perforating arteries, and its usefulness has been reported [1–3]. Recently, the detectability of low-speed blood flow employing the Doppler method has markedly improved with advancement in ultrasonographic diagnostic devices, and the accuracy has also markedly improved. In particular, the introduction of the power Doppler method, which is excellent for detecting low velocity blood flow, has allowed the visualization of peripheral vessels, which was conventionally difficult, and its application has expanded to many departments.

**Figure 4:**
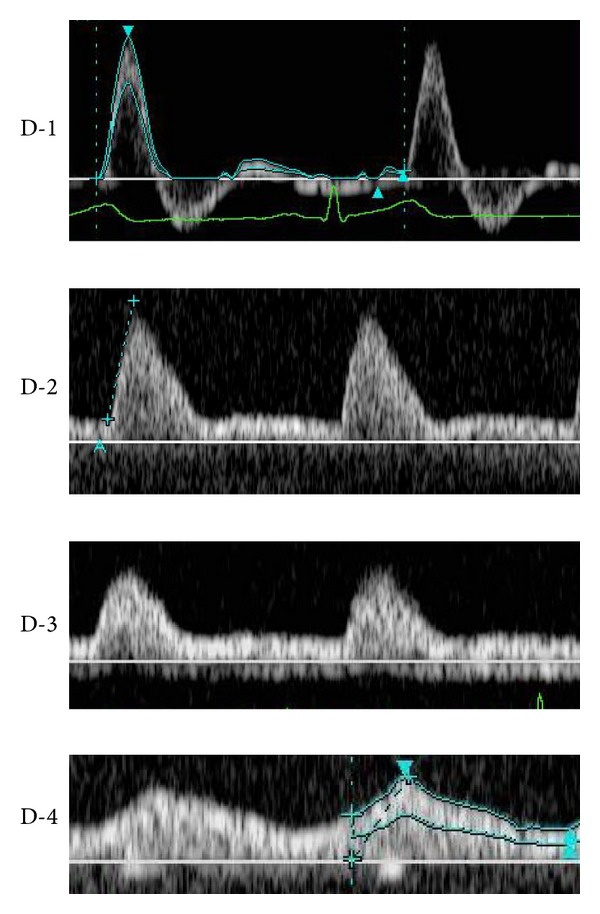
Hirai's classification of the vascular waveform. Hirai classified vascular waveforms into 4 types (D-1 to D-4).

**Figure 5:**
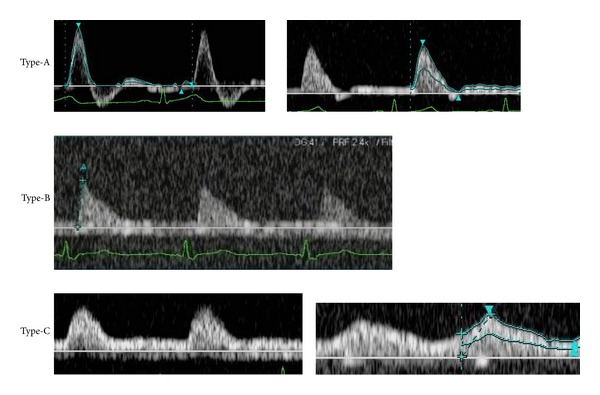
Baba's classification of the vascular waveform. Baba classified vascular waveforms into 3 types: type A: systolic waves rise steeply and are followed by reflux components in diastole, type B: the waveform is comprised of only systolic waves and diastolic waves are lost, and type C: systolic waves rise slowly and continue antegradely in diastole.

Regarding vascular waveform analysis of peripheral blood vessels, analysis is applied to diagnose lower limb occlusive arterial diseases. Hirai et al. [5] applied it for the screening of pelvic and lower limb occlusive arterial diseases, and classified the vascular waveforms into 4 types: D-1 to D-4 (Figure 4). Baba et al. [8] performed vascular waveform analysis of severely ischemic lower limbs and observed that whether the waveform type is D-1 or not could be determined following the waveform classification reported by Hirai et al.; but classification of the other types was difficult in many cases. They classified waveforms based on the presence or absence of diastolic components and direction (antegrade/retrograde) into the following 3 types: Type A to C (Figure 5). However, some arterial waveforms were difficult to classify employing the classifications reported by Hirai et al. and Baba et al. in our patients: systolic waves rose steeply but were not followed by reflux components in diastole, a notch was present between the systolic and diastolic waves, and anterograde diastolic waves were observed. No reflux component was noted in diastolic waves in many cases, even though the vascular resistance was high and systolic waves showed a favorable steep rise in flap-feeding arteries, because these are peripheral arteries with a smaller diameter than those of the distal external iliac artery and proximal region of the popliteal artery involved in the waveform classification reported by Hirai et al. Thus, we considered it inappropriate to classify all these waveforms as D-2 and classified them as D-1b when a notch was noted between systolic and diastolic waves and designated the original D-1 as D-1a (Figure 1).

Hirai et al. regarded the D-1 type as a normal waveform with no significant stenosis on the proximal side and suggested the presence of a stenosis lesion on the proximal side in D-2, D-3, and D-4 waveforms. However, these are applied to arterial waveforms in the pelvis and thighs but difficult to apply to peripheral arteries unless some modification is made. In peripheral arteries, diastolic waveforms are lost because peripheral vascular resistance is high, and *V*_min⁡_ is nearly 0 in many waveforms. Since these waveforms may also be normal in peripheral arteries, we regarded D-1a, D-1b, and D-2 as normal waveforms in the classification.

As parameters for the quantification of pulsative waveforms, based on *V*_max⁡_, *V*_min⁡_, and *V*_mean_, the A/B ratio calculated as *V*_max⁡_/*V*_min⁡_, RI as (*V*_max⁡_ − *V*_min⁡_)/*V*_max⁡_, PI as (*V*_max⁡_ − *V*_min⁡_)/*V*_mean_, and AT have been used. High and low values of A/B ratio, RI, and PI indicate high and low peripheral vascular resistances, respectively. The A/B ratio and RI are simply calculated from *V*_max⁡_ and *V*_min⁡_, and the reproducibility is superior because values with relatively small errors are used, showing that these are practical parameters. However, the A/B ratio reaches infinity when *V*_min⁡_ comes close to 0, and the RI can be calculated even when peripheral vascular resistance is markedly high and no diastolic blood flow component is observed in waveforms, but the value becomes RI = 1 regardless of the waveform shape. On the other hand, the PI value has no problem because of the use of *V*_mean_ even when no diastolic blood flow component is observed, but the calculation is not simple because the mean flow rate is calculated by tracing blood flow waveforms. Moreover, its calculation accuracy is inferior to that of RI due to technical errors. AT is an index reflecting changes in the time axis. Its calculation is simple, and an element in the time axis direction is added, for which improvement of the accuracy of blood flow evaluation is expected. In this study, we used RI, PI, and AT as indices for quantification of the pulsating waveforms of blood flow.

We performed vascular waveform analysis of the flap-feeding vessels of about 40 patients and reported the established reference values based on measurement values of vascular diameter, F-V, RI, PI, and AT [4] (Table 2). Now it is applied as an index of the blood flow in the flaps prior to surgery. Although there is some difference by the kind of flap-feeding vessels about the standard value for judged whether the transplantation of the skin flap is safe; vascular diameter ≧ 1 mm; *FV*≧5 mL/min; *V*_*max*≧15 cm/sec; *RI*≧0.7; and *AT*≦100 msec.

On a comparison of the results of vascular waveform analysis in actual cases of flap transplantation with the established standard values, the vascular waveform were normal (D-1a or D-1b or D-2) in cases which achieved complete survival, the diameter, FV, and *V*_max⁡_, were higher than the established values, indicating favorable blood flow, and the RI, PI, and AT were within the standard value ranges.

On the other hand, an abnormal D-4 waveform was noted in a case in which superior epigastric arterial waveform analysis was performed before surgery for sternal osteomyelitis. The *V*_max⁡_ was 15.6 cm/sec, being slower than the standard; RI was 0.34, and PI was 0.50, being lower than the standard (Figure 2). The surgical procedure was changed to one using lattisimus dorsi musculocutaneous flap, and it was possible to perform a safe operation. In the case in which the pedicled flap containing the superior epigastric artery was partially necrosed, the waveform was abnormal D-3, and the *V*_max⁡_ was 15.0 cm/sec, being lower than the standard (Figure 3).

The case which detected blood flow poorly, the vascular obstruction of the internal thoracic artery, was able to be judged prior to surgery, and it was possible to perform safe operation by changing into the reconstruction using omentum and pectoralis major muscle flap. The above clinical findings reflected the usefulness of the established standards.

The following advantages can be mentioned in the color Doppler ultrasonography. (1) It can be visualized the vessels at the same time as the fascia and muscle, adipose tissue, bone, it is possible to understand the detailed running of flap-feeding vessels. (2) We can select the skin flap with good blood flow more by measuring vessel diameter, flow volume, and flow velocity. (3) In the free flap transplant patients, postoperative patency confirmation of vascular anastomosis is easy; it can be observed noninvasively and frequent times. (4) Therefore, it is useful for early detection of vascular occlusion such as postoperative thrombosis. (5) Quantitative observation of blood flow velocity of the anastomotic vessels after surgery is possible. However, technical skills, such as the skill of acquiring vascular images and experience in device operation are necessary. To perform vascular waveform analysis by visualizing the vascular distribution in detail and measuring the vascular diameter, F-V, RI, PI, and AT, collaboration with technologists is necessary, which will take time to conduct tests to some extent. To identify the distribution of a flap-feeding vessels and detailed hemodynamics, that is, for preoperative evaluation to perform safe surgery, it may be necessary for an operator familiar with vascular anatomy to attend to the tests and collaboration with technologists for the operation and setting of the ultrasonography system.

## 5. Conclusions

As preoperative blood flow assessment of the flap-feeding vessels, we performed vascular waveform analysis using color Doppler ultrasonography. Based on arterial waveform of the flap-feeding vessels, F-V, RI, PI, and AT were measured and classified vascular waveforms into 5 types partially modifying the blood flow waveform classification of Hirai et al. Although skills are necessary for the manipulation and setting of the ultrasonography system, and collaboration with clinical technologists is required, it is important to evaluate not only basic blood flow information of the vascular distribution, vascular diameter, and flow velocity, but also the flow volume, vascular resistance, and arterial waveform in analyzing the hemodynamics of the vascularised flaps and essential in performing Doppler ultrasonography.

## Figures and Tables

**Table 1:** Clinical characteristics of patients. Vascular waveform analysis of thoracodorsal artery was performed in 8 patients, deep inferior epigastric artery in 2 patients, superior epigastric artery in 6 patients, thoracoacrominal artery in 3 patients, and lateral circumflex femoral artery in 2 patients.

Patient	Age	Sex	Disease	Selected flap	Vascular waveform analysis
1	67	M	Mediastinitis	LDMC	SEA and TDA
2	36	F	Breast cancer	LDMC	TDA
3	33	F	Breast cancer	LDMC	TDA
4	59	M	Esophageal fistula	LDMC	TDA
5	44	F	Breast cancer	LDMC	TDA
6	41	F	Breast cancer	LDMC	TDA
7	55	F	Scar contracture after burn	LDMC	TDA
8	79	F	Mediastinitis	LDMC	TDA
9	55	M	Mediastinitis	RAMC	SEA
10	78	F	Mediastinitis	RAM	SEA
11	69	M	Rectal cancer anal invasion	RAMC	DIEA
12	67	M	Mediastinitis	RAMC	SEA
13	67	F	Vaginal defect	RAMC	DIEA
14	48	F	Breast cancer	RAMC	SEA
15	68	M	Esophageal fistula	PMMC	TAA
16	67	M	Mediastinitis	PMMC	TAA
17	57	M	Mediastinitis	PMM	SEA & TAA
18	71	M	Gallbladder cancer abdominal wall invasion	TFLMC	LCFA
19	71	F	Abdominal wall incisional herniatis	TFLMC	LCFA

LDMC: lattisimus dorsi musculocutaneous flap; RAMC: rectus abdominis musculocutaneous flap; RAM: rectus abdominis muscle flap; PMMC: pectoralis major musculocutaneous flap; PMM: pectoralis major muscle flap; TFLMC: tensor fascia lata musculocutaneous flap; TDA: thoracodorsal artery; SEA: superior epigastric artery; DIEA: deep inferior epigastric artery; TAA: thoracoacrominal artery; LCFA: lateral circumflex femoral artery.

**Table 2:** Reference values of epigastric artery and subscapular-thoracodorsal artery. The standard values for the superior epigastric artery were: vascular diameter, 0.8 mm or greater; FV, 7 mL/min or faster; *V*_max⁡_, 15 cm/sec or greater; RI, 0.7 or greater; PI, 2.2 ± 0.8; and AT, ≦100 msec. Those for the deep inferior epigastric artery were: vascular diameter, 1.3 mm or greater; FV, 10 mL/min or faster; *V*_max⁡_, 25 cm/sec or greater; RI, 0.7 or greater; PI, 2.7 ± 1; and AT, ≦100 msec. Those for the subscapular artery were: vascular diameter, 2 mm or greater; FV, 20 mL/min or faster; *V*_max⁡_, 30 cm/sec or greater; RI, 0.7 or greater; PI, 4 ± 2; and AT, ≦100 msec. Those for the thoracodorsal artery were: vascular diameter, 1 mm or greater; FV, 5 mL/min or faster; *V*_max⁡_, 20 cm/sec or greater; RI, 0.7 or greater; PI, 4 ± 2; and AT, ≦100 msec.

	Superior epigastric artery (*N* = 9)	Deep inferior epigastric artery (*N* = 20)	Subscapular artery (*N* = 12)	Thoracodorsal artery (*N* = 12)
Diameter (mm)	0.8 mm or more	1.3 mm or more	2 mm or more	1 mm or more
FV (mL/min)	7 mL/min or more	10 mL/min or more	20 mL/min or more	5 mL/min or more
*V*_max⁡_ (cm/sec)	15 cm/sec or more	25 cm/sec or more	30 cm/sec or more	20 cm/sec or more

RI	0.7 or more	0.7 or more	0.7 or more	0.7 or more
PI	2.2 ± 0.8	2.7 ± 1	4 ± 2	4 ± 2
AT	≦100 msec	≦100 msec	≦100 msec	≦100 msec

**Table 3:** Vascular waveform analysis of flap-feeding vessels. They were 4 patients which observed abnormal vascular waveform among 19 patients which performed vascular waveform analysis of flap-feeding vessels by using color Doppler ultrasonography prior to surgery (D-4: 1 patient, D-3: 1 patient, and Poor detect: 2 patients).

Patient	Vascularity	Side	Diameter	FV	*V*_max⁡_	*V*_min⁡_	*V*_mean_	RI	PI	AT	Wave form
1	SEA	Lt	1.3 mm		15.6 cm/sec	10.3 cm/sec	10.6 cm/sec	0.34	0.50	125 msec	D-4
	TDA	Rt	1.6 mm		25.6 cm/sec	2.9 cm/sec	10.4 cm/sec	0.81	1.98	92 msec	D-2
2	TDA	Lt	1.3 mm	20 mL/min	35.3 cm/sec	6.8 cm/sec	14.2 cm/sec	0.81	2.01	66 msec	D-1b
3	TDA	Rt	2.0 mm	14 mL/min	26.0 cm/sec	2.6 cm/sec	7.6 cm/sec	0.90	3.08	25 msec	D-1a
4	TDA	Lt	1.3 mm	6 mL/min	23.5 cm/sec	2.8 cm/sec	7.1 cm/sec	0.81	2.68	58 msec	D-1b
5	TDA	Rt	0.7 mm	2 mL/min	22.6 cm/sec	0 cm/sec	6.6 cm/sec	1.00	3.42	50 msec	D-2
6	TDA	Rt	1.8 mm	14 mL/min	56.5 cm/sec	0 cm/sec	9.4 cm/sec	1.00	6.01	54 msec	D-1a
7	TDA	Rt	2.3 mm	37 mL/min	53.3 cm/sec	4.4 cm/sec	14.8 cm/sec	0.92	3.30	58 msec	D-1b
8	TDA	Rt	1.7 mm	20 mL/min	40.5 cm/sec	4.6 cm/sec	14.6 cm/sec	0.89	2.46	63 msec	D-1b
9	SEA	Lt	1.1 mm		24.3 cm/sec	4.7 cm/sec	10.1 cm/sec	0.81	1.94	62 msec	D-1b
10	SEA	Rt	2.1 mm	12 mL/min	15.0 cm/sec	2.3 cm/sec	5.7 cm/sec	0.85	2.23	75 msec	D-3
11	DIEA	Rt	1.8 mm	15 mL/min	42.3 cm/sec	1.2 cm/sec	12.9 cm/sec	0.87	2.86	71 msec	D-1b
12	SEA	Lt	1.0 mm	4 mL/min	19.1 cm/sec	4.4 cm/sec	7.6 cm/sec	0.81	2.04	71 msec	D-2
13	DIEA	Lt	1.3 mm	27 mL/min	81.1 cm/sec	16 cm/sec	34 cm/sec	0.8	1.91	45 msec	D-1b
14	SEA	Lt	1.3 mm	7 mL/min	25.8 cm/sec	3.6 cm/sec	9.7 cm/sec	0.86	2.29	46 msec	D-2 (ITA: poor detect)
15	TAA	Rt	1.8 mm	21 mL/min	45.5 cm/sec	4.7 cm/sec	13.9 cm/sec	0.94	3.07	62 msec	D-2
16	TAA	Lt	2.2 mm	19 mL/min	51.1 cm/sec	5.9 cm/sec	8.4 cm/sec	0.88	5.38	58 msec	D-1b
17	TAA	Lt	1.9 mm	28 mL/min	51.5 cm/sec	4.9 cm/sec	9.1 cm/sec	0.87	2.69	62 msec	D-1b (SEA: poor detect)
18	LCFA	Rt	1.7 mm	18 mL/min	37.9 cm/sec	6.3 cm/sec	13.4 cm/sec	0.74	2.10	70 msec	D-2
19	LCFA	Rt	1.7 mm	12 mL/min	32.4 cm/sec	2.4 cm/sec	8.8 cm/sec	0.93	3.41	54 msec	D-1b

TDA: thoracodorsal artery; SEA: superior epigastric artery; DIEA: deep inferior epigastric artery; TAA: thoracoacrominal artery; LCFA: lateral circumflex femoral artery.

**Table 4:** Vascular waveform classification and survival of the transplanted musculocutaneous flaps. For the survival of the transplanted musculocutaneous flaps, two cases of superior epigastric artery pedicled rectus abdominis musculocutaneous flaps were completely survived in four cases, two cases were partial necrosis. Two cases of deep inferior epigastric artery pedicled rectus abdominis musculocutaneous flaps were completely survived. Eight cases of thoracodorsal artery pedicled latissimus dorsi musculocutaneous flaps were completely survived. Three cases of thoracoacrominal artery pedicled pectoralis major musculocutaneous flaps were completely survived. Two cases of lateral circumflex femoral artery pedicled tensor fascia lata musculocutaneous flaps were completely survived.

	Superior epigastric artery		Deep inferior epigastric artery		Thoracodorsal artery		Thoracoacrominal artery		Lateral circumflex feroral artery	
	Number	Outcome	Number	Outcome	Number	Outcome	Number	Outcome	Number	Outcome
D-1a	0		0		3	S: 3	0		0	
D-1b	1	S	1	S	3	S: 3	3	S: 3	1	S
D-2	2	S: 1 PN: 1	1	S	2	S: 2	0		1	S
D-3	1	PN: 1	0		0		0		0	
D-4	0		0		0		0		0	

PN: partial necrosis; S: survive.
